# Avatar Mice Underscore the Role of the T Cell-Dendritic Cell Crosstalk in Ebola Virus Disease and Reveal Mechanisms of Protection in Survivors

**DOI:** 10.1128/jvi.00574-22

**Published:** 2022-09-08

**Authors:** Monika Rottstegge, Tom Tipton, Lisa Oestereich, Paula Ruibal, Emily V. Nelson, Catherine Olal, Julia R. Port, Johan Seibel, Elisa Pallasch, Sabrina Bockholt, Fara Raymond Koundouno, Joseph Akoi Boré, Estefanía Rodríguez, Beatriz Escudero-Pérez, Stephan Günther, Miles W. Carroll, César Muñoz-Fontela

**Affiliations:** a Bernhard Nocht Institute for Tropical Medicine, Hamburg, Germany; b German Center for Infection Research (DZIF) Partner Site Hamburg-Lübeck-Borstel-Riems, Hamburg, Germany; c Pandemic Sciences Institute & Wellcome Centre for Human Genetics, University of Oxford, United Kingdom; d Department of Infectious Diseases, Leiden University Medical Center, Leiden, The Netherlands; e University Hospital Hamburg-Eppendorf, Institute for Transfusion Medicine, Hamburg, Germany; f Laboratoire du Projet des Fièvres Hémorragiques de Guinée (PFHG), Conakry, Guinea; The Peter Doherty Institute for Infection and Immunity

**Keywords:** Ebola virus, T cells, dendritic cells, humanized mice, avatar, Ebola, mouse models

## Abstract

Ebola virus disease (EVD) is a complex infectious disease characterized by high inflammation, multiorgan failure, the dysregulation of innate and adaptive immune responses, and coagulation abnormalities. Evidence accumulated over the last 2 decades indicates that, during fatal EVD, the infection of antigen-presenting cells (APC) and the dysregulation of T cell immunity preclude a successful transition between innate and adaptive immunity, which constitutes a key disease checkpoint. In order to better understand the contribution of the APC-T cell crosstalk to EVD pathophysiology, we have developed avatar mice transplanted with human, donor-specific APCs and T cells. Here, we show that the transplantation of T cells and APCs from Ebola virus (EBOV)-naive individuals into avatar mice results in severe disease and death and that this phenotype is dependent on T cell receptor (TCR)-major histocompatibility complex (MCH) recognition. Conversely, avatar mice were rescued from death induced by EBOV infection after the transplantation of both T cells and plasma from EVD survivors. These results strongly suggest that protection from EBOV reinfection requires both cellular and humoral immune memory responses.

**IMPORTANCE** The crosstalk between dendritic cells and T cells marks the transition between innate and adaptive immune responses, and it constitutes an important checkpoint in EVD. In this study, we present a mouse avatar model in which T cell and dendritic cell interactions from a specific donor can be studied during EVD. Our findings indicate that T cell receptor-major histocompatibility complex-mediated T cell-dendritic cell interactions are associated with disease severity, which mimics the main features of severe EVD in these mice. Resistance to an EBOV challenge in the model was achieved via the transplantation of both survivor T cells and plasma.

## INTRODUCTION

Ebola virus disease (EVD) is caused by infection with viruses of the genus *Ebolavirus*, most notably *Zaire ebolavirus* (EBOV), which is responsible for most of the outbreaks reported to date, including the West Africa EVD epidemic of 2013 to 2016 and the recent outbreaks in Guinea and in the Democratic Republic of Congo (DRC) (1). EVD is a complex disease with high case-fatality rates that involves the dysregulation of many physiological processes, including inflammation, immunity, coagulation, electrolyte balance, and endothelial activation. In particular, the disruption of the host immune response seems to be an important aspect of EVD pathophysiology. In both human and animal models, fatal EBOV infection is associated with many hallmarks of immune failure, such as infection, the loss of circulating antigen-presenting cells (APCs), the presence of “exhausted” or hyperactivated T cells in the polyclonal repertoire, the loss of T cell clonal diversity, and, in some instances, the poor formation of EBOV-specific T cells and antibodies (2).

Many of these immune defects seem to revolve around the transition between innate and adaptive immunity, an important checkpoint to mounting host defenses against emerging viruses for which most of the population do not have a preexisting antibody immunity. Indeed, many previous studies have shown that EBOV has evolved mechanisms by which to antagonize the function of dendritic cells (DCs), which are professional APCs with a chief role in the activation of cognate naive T cells (3, 4). Through their ability to sense antigens in peripheral tissues such as the mucosae and skin, DCs are uniquely suited to initiate pathogen-specific immune responses. After antigen recognition, DCs can travel via lymphatics to tissue-draining lymph nodes, encounter cognate naive T cells, and provide the necessary signals for T cell activation (5).

A common feature of highly pathogenic viruses is the induction of high levels of proinflammatory mediators in the host. Many emerging viruses, in particular, zoonotic RNA viruses, cause severe diseases that are characterized by exacerbated inflammation in humans. Clinically, these infections may be different, such as hemorrhagic fever in the case of systemic infections or acute respiratory distress syndrome (ARDS) in respiratory infections (6). However, the underlying cause of severity is uncontrolled inflammation, which is linked to a defective transition from innate to adaptive immunity in the absence of preexisting virus-specific antibodies.

The loss of APC function in emerging viral infections has been linked to type I interferon (IFN-I) antagonism. IFN-I signaling is necessary for DC maturation and activation (7). Through their ability to encode IFN-I antagonist proteins, many emerging viruses prevent the activation of DCs, which allows virus replication to proceed at an early stage after infection (8, 9). Many studies in animal models have demonstrated that, after this initial phase, high levels of virus replication trigger late inflammatory responses resulting in T cell overactivation and a cytokine storm, leading to the further activation of bystander T cells (10, 11). These findings strongly suggest that the crosstalk between DCs and T cells is at the center of the pathophysiology of viral hemorrhagic fevers, including EVD. In this study, we sought to set up an *in vivo* system to test this hypothesis.

In previous studies, we have shown that severely immunodeficient mice engrafted with human hematopoietic stem cells (human immune system or HIS mice), are highly susceptible to EBOV infection (12) and recapitulate the case-fatality rates of ebolaviruses in humans (13). While this model is well suited for pathogenesis studies as well as for the testing of medical countermeasures, it does not adequately reflect human immune responses to EBOV infection due to limited T cell responses and the lack of immunoglobulin isotype switching.

To study the interaction of human DCs and T cells, one possibility is the use of avatar mouse models. The engineering of avatar mice is based on the principle of transplantation of donor-specific xenografts into immune-suppressed mice. In tumor biology, for example, this strategy has been used to devise personalized medicine approaches via the transplantation of tumors that retain the histopathological, genetic, and epigenetic information of the donor (14). While the interest in mouse avatars as cancer models is increasing significantly, to the best of our knowledge, this approach has not been used for research on emerging infectious diseases. One advantage of the avatar mouse model approach is that, as opposed to conventional humanized immune system (HIS) mice, avatar mice conserve the immune memory information of the donor and could potentially serve to correlate the presence of donor-specific T cells with protection.

Here, we have used the sequential transplantation of lymphoid and myeloid peripheral blood cells from individual donors into HLA-A2-transgenic NOD-*scid*-interleukin 2 receptor-γ knockout mice (NSG-A2) to study the relevance of the DC-T cell crosstalk during EBOV infection. In these avatar mice, reconstitution with peripheral blood cells from EBOV-naive individuals followed by the adoptive transfer of autologous DCs infected with EBOV *ex vivo* resulted in lethality, which was dependent on TCR-HLA interactions. The transplantation of both T cells and plasma from EVD survivors resulted in avatar mice that survived an EBOV challenge. Our findings provide an *in vivo* model suitable to study the correlates of protection to EBOV and possibly those of other emerging viruses.

## RESULTS

### Generation of avatar mice.

The engraftment of severely immune deficient mice with human immune cells is a common strategy by which to study immune cell functions during infectious diseases or several other inflammatory processes. The most common strategy is the reconstitution of human hematopoiesis in any of the multiple versions of NOD-*scid* mice via the transplantation of hematopoietic stem cells. We have, for example, used this strategy previously to study filovirus pathogenesis (12, 13). However, a limitation of this model is that progenitor cell-based reconstitution results in an immune system that is generated *de novo*. Therefore, all immune memory information from the donor is lost. In order to study the importance of the T cell-DC crosstalk on EBOV pathogenesis, we decided to engraft HLA-A2 transgenic NOD-*scid*-interleukin (IL)-2 receptor gamma knockout mice (NSG-A2) with peripheral blood leukocytes from HLA-A2^+^ donors.

**FIG 1:**
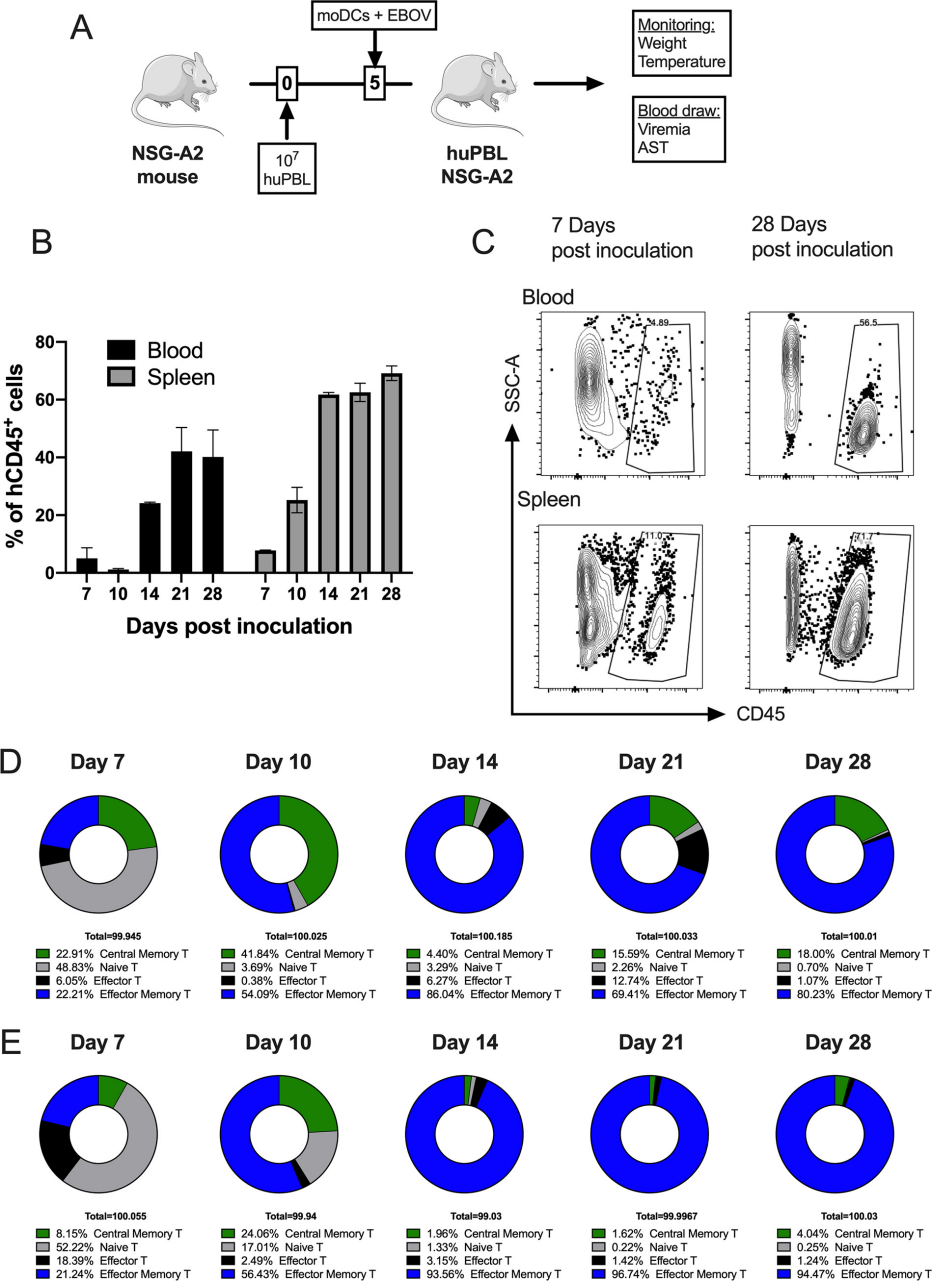
Generation and characterization of avatar mice. (A) Schematic indicating the procedure by which to generate mouse avatars. HuPBLs (CD14 negative PBMC fraction) are transplanted at day 0. CD14^+^ cells from the same donor are incubated with GM-CSF and IL-4 to generate immature monocyte-derived DCs, which are then infected with EBOV at an MOI of 1 and transplanted at day 5. (B) Frequency of human hematopoietic cells (hCD45^+^) in the peripheral blood of avatar mice at the indicated time points. Data are shown as the mean ± the standard error the mean (SEM). (C) Representative flow cytometry plots of the data shown in panel B. (D) Frequency of CD8^+^ central memory, effector memory, naive and effector T cells (TEMRA) at the indicated time points in the peripheral blood of avatar mice, as assessed by flow cytometry. (E) Frequency of CD4^+^ central memory, effector memory, naive and effector T cells (TEMRA) at the indicated time points in the peripheral blood of avatar mice, as assessed by flow cytometry.

Our strategy was based on a protocol previously described by Harui et al. (15), which relies on the sequential transplantation of peripheral blood lymphocytes (huPBL) and donor-specific DCs. Before transplantation, we separated huPBL and myeloid cells in donor peripheral blood using the positive selection of CD14^+^ cells. This strategy results in two cell fractions, the CD14^+^ monocytes and the CD14-huPBL fraction, in which T cells accounted for 87% of the CD45^+^ leukocyte population (Fig. S1A). HuPBL cells were transplanted into recipient NSG-A2 mice at day 0. At the same day, CD14^+^ cells were seeded in culture in the presence of human granulocyte-macrophage colony-stimulating factor (GM-CSF) and IL-4 to obtain monocyte-derived DCs (moDCs) (Fig. S1B). Five days after the transplantation of huPBL, derived moDCs were infected with EBOV or mock-infected *ex vivo* and transplanted (0.5 to 1 × 10^6^) into recipient avatar mice (Fig. 1). As previously described (15), human CD45^+^ leukocytes populated the mouse peripheral blood and spleen during the next 3 weeks such that at day 28, 60% of the leukocytes in the spleen were of human origin (Fig. 1B and C). As indicated by Harui et al., the analysis of the T cell fraction showed a progressive skewing of T cells toward an effector/memory phenotype (T_EM_), so that T_EM_ cells were dominant at days 21 to 28 after transplantation (Fig. 1D and E).

### Donor-specific responses to EBOV infection in avatar mice.

**FIG 2:**
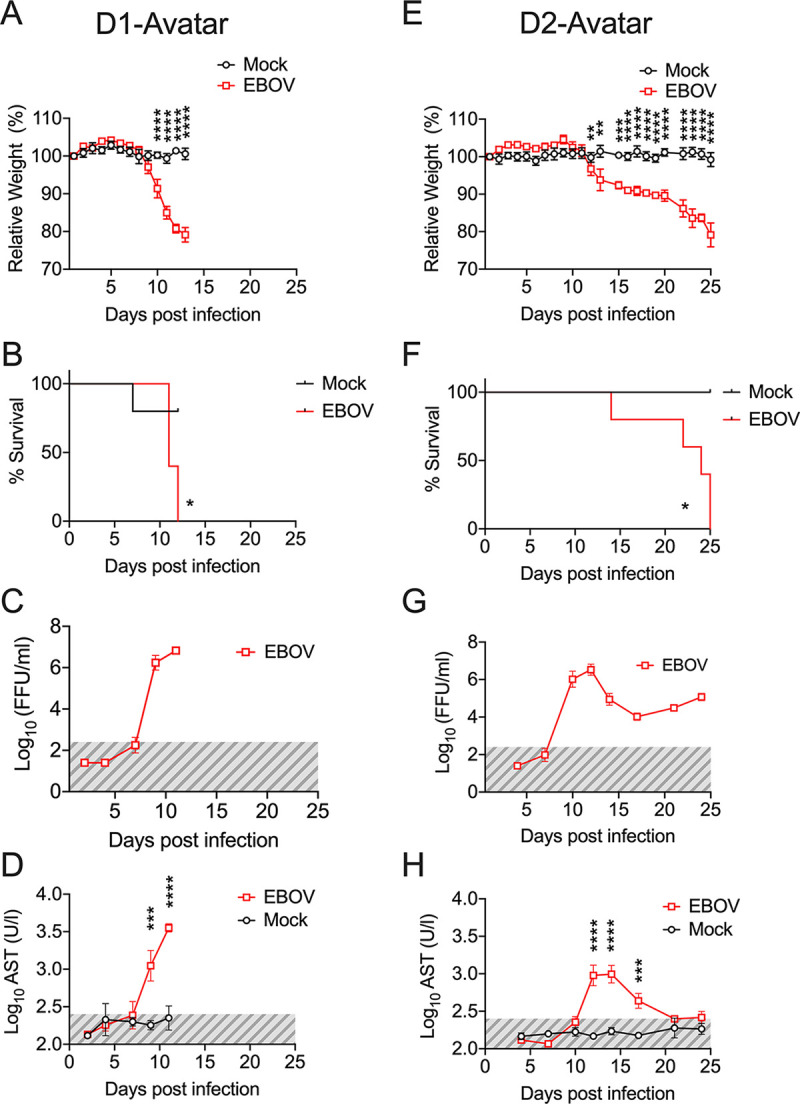
The EVD course in avatar mice is donor-specific. (A and E) Body weight loss curves of avatar mice transplanted with huPBL and DCs from donor 1 (D1) and donor 2 (D2), respectively. Mock-mice were transplanted with noninfected DCs, whereas EBOV-mice were transplanted with DCs infected with EBOV for 60 min at an MOI of 1. (B and F) Kaplan-Meier survival curves for D1 and D2-avatar mice. (C and G) Levels of viremia as determined by the assessment of EBOV infectious particles via focus-formation assay at the indicated time points. Shaded areas (gray) mark the limit of detection of the assay. (D and H) Levels of serum aspartate transaminase (AST) in D1 and D2 avatars. In panels A, E, D, and H, statistical significance was determined using a two-way analysis of variance (ANOVA) followed by Dunnett’s multiple comparison test. In panels B and F, statistical evaluation was performed via the Mantel-Cox test. Across the figure, significance levels are presented as follows: *, *P* ≤ 0.05; **, *P* ≤ 0.01; ***, *P* ≤ 0.001, ****; and *P* ≤ 0.0001. Data are shown as mean ± SEM. D1 (Mock *n* = 5, EBOV *n* = 5), D2 (Mock *n* = 5, EBOV *n* = 4).

To initially characterize the response of the avatar mice to EBOV infection, we generated two groups of mice engrafted with huPBL and moDCs from two different EBOV naive HLA-A2^+^ donors (D1 and D2). In both groups (D1-avatar and D2-avatar), five mice were transplanted with EBOV-infected moDCs, while control mice (*n* = 5 for D1 and *n* = 4 for D2) were transplanted with mock-infected moDCs. EBOV-infected D1-avatar mice lost weight rapidly and showed high levels of viremia, reaching 10^6^ FFU/mL, and high levels of circulating serum aspartate aminotransferase (AST). 100% of the mice died within the first 2 weeks after infection (Fig. 2A to D). Necropsy findings in this group of mice revealed the presence of gastrointestinal bleeding and liver steatosis (Fig. S2). The D2-avatar mice also showed signs of severe disease with progressive weight loss and 100% lethality, but this group showed a delayed time of death, with most subjects reaching the euthanasia criterion (i.e., weight loss) at 3 weeks after infection (Fig. 2E and F). In this group, two peaks of viremia were observed at days 12 and 25 after infection, and the levels of serum AST were substantially lower than those in the D1-avatars (Fig. 2G and H).

These results indicated that avatar mice may be able to serve in the investigation of donor-specific responses to EBOV infection.

### EBOV-induced disease in avatar mice requires HLA-dependent T cell-DC interactions.

DCs are primary targets of EBOV infection, and migratory DCs are likely responsible for virus dissemination from the initial infection sites to the body (11). The use of avatar mice allows us to modify the T cell-DC crosstalk to further investigate how interactions between these immune cells influence EBOV pathogenesis.

**FIG 3:**
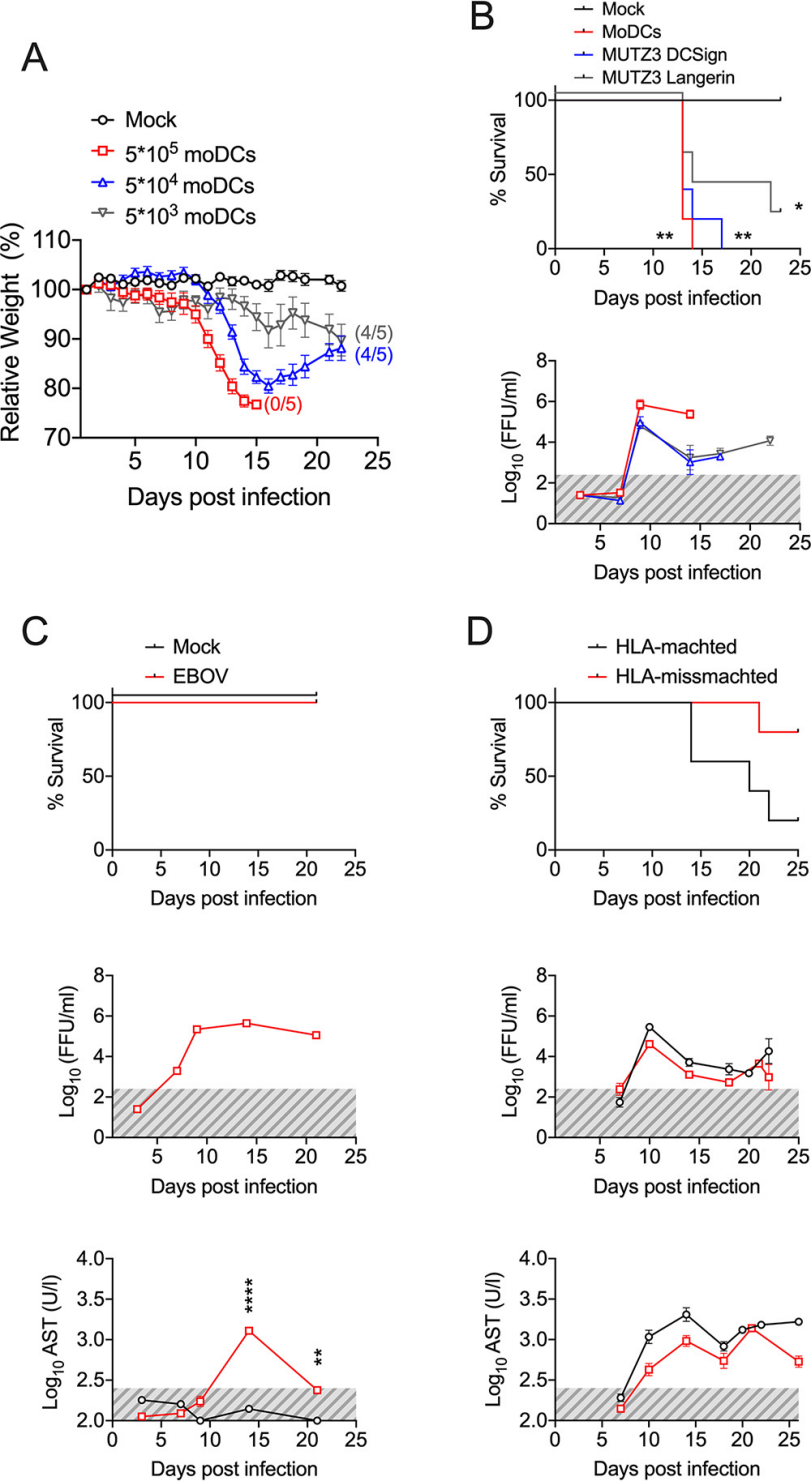
The onset of EVD in avatar mice depends on T cell-DC interactions. (A) Body weight loss curves of avatar mice transplanted with EBOV-infected DCs at the indicated inputs. Numbers between brackets indicate the number of surviving mice out of *n* = 5 mice in each group. (B) The Kaplan-Meier survival curves of avatar mice transplanted with infected DCs are as follows. Mock-mice were transplanted with noninfected moDCs, and moDCs indicates monocyte-derived DCs. MUTZ3-DCSign indicates MUTZ-3 cells derived into DC populations enriched in interstitial DC-Sign-like DCs. MUTZ3-Langerin indicates MUTZ-3 cells derived into DC populations enriched in Langerhans-like cells. The bottom panel indicates viremia, as assessed via focus forming assay at the indicated time points. This experiment was done once with *n* = 5 mice/group. (C) Avatar mice were transplanted with EBOV-infected moDCs in the absence of a previous infusion of autologous huPBLs. The Kaplan-Meier survival curves in comparison with the mock are shown in the upper panel, while the viremia and AST serum levels are shown in the lower panels. Gray areas indicate the limit of detection. (D) Avatar mice were generated by the transplantation of huPBLs followed by the transplantation of EBOV-infected moDCs from HLA-matched (HLA-A*02:01) or mismatched donors. Survival curves, viremia and AST levels in serum are shown. Across the figure, significance levels are presented as follows: *, *P* ≤ 0.05; **, *P* ≤ 0.01; ***, *P* ≤ 0.001; and ****, *P* ≤ 0.0001. The survival curves were assessed via Mantel-Cox. All other analyses were conducted with a two-way ANOVA followed by Dunnett’s multiple comparison test. Data are shown as mean ± SEM.

We reasoned that if DCs supported early EBOV replication and dissemination, we could alleviate disease manifestations by reducing the input of infected DCs. Indeed, whereas in the mice transplanted with 5 × 10^5^ moDCs, all of the animals lost weight rapidly and succumbed to infection within 2 weeks postinoculation, in the mice transplanted with 5 × 10^4^ or 5 × 10^3^ moDCs, four out of five mice survived. In the group with 5 × 10^3^ moDCs, we also observed a substantial reduction of morbidity, namely, the weight loss and body scoring parameters (Fig. 3A).

DCs are a complex group of immune cells that differ in their functions as antigen-presenting cells, their localization in the body, and their bone marrow lineages (5, 16). Although moDCs are widely studied as a general DC model *in vitro*, we wanted to address whether our findings were applicable to other DC subsets. To do so, we took advantage of the plasticity of the HLA-A2^+^ MUTZ-3 cell line, which behaves as the immortalized equivalent of CD34^+^ DC precursor cells (17). Thus, using different cytokine cocktails in cell culture (see Materials and Methods), we differentiated MUTZ-3 cells into interstitial-like DC-SIGN^+^ DCs or Langerhans cell-like Langerin^+^ DCs. We then utilized the same protocol described for the donor-derived moDCs to infect differentiated MUTZ-3 cells with EBOV and transplant them into avatar mice harboring HLA-A2^+^-matched huPBL. As described for the moDC avatar model, mice transplanted with MUTZ-3 DC-SIGN or MUTZ3 Langerin EBOV-infected cells lost weight rapidly, reached high levels of viremia, and succumbed to infection (Fig. 3B). These results indicated that the avatar model was not dependent on the specific subtype of transplanted DC.

Since the infected DCs seemed to be the main modulators of disease severity in the avatar mice, we then wondered whether the transplantation of infected DCs alone could be sufficient to recapitulate disease in the model. To test this hypothesis, we transplanted 5 × 10^5^ EBOV-infected moDCs or their mock-infected counterparts into NSG-A2 mice which had not been previously infused with huPBL. Mice developed significant levels of viremia and showed elevated levels of serum AST that peaked at day 15 posttransplantation. However, none of the mice showed any signs of disease, and all of the mice survived (Fig. 3C). These findings demonstrated that both infected DCs and donor T cells are required in order to recapitulate EVD in the avatar model.

Antigen presentation from DCs to T cells requires interactions between HLA molecules and the T cell receptor (TCR). In order to test the importance of the HLA-TCR interactions for EBOV-induced disease in the avatar mice, we compared disease in mice with HLA-matched DCs and T cells as well as in mice in which the T cells and DCs were from donors with mismatched HLAs. Despite similar levels of viremia and elevated serum ASTs, 80% of the HLA-TCR mismatched avatar mice (4 out of 5) survived EBOV infection (Fig. 3D). Taken together, our findings indicate that in the avatar mice, lethal EBOV infection requires the transplantation of both T cells and DCs and that the severity of the model is dependent on HLA-TCR interactions.

### Avatar mice recapitulate ebolavirus pathogenesis but not Lassa virus pathogenesis.

**FIG 4:**
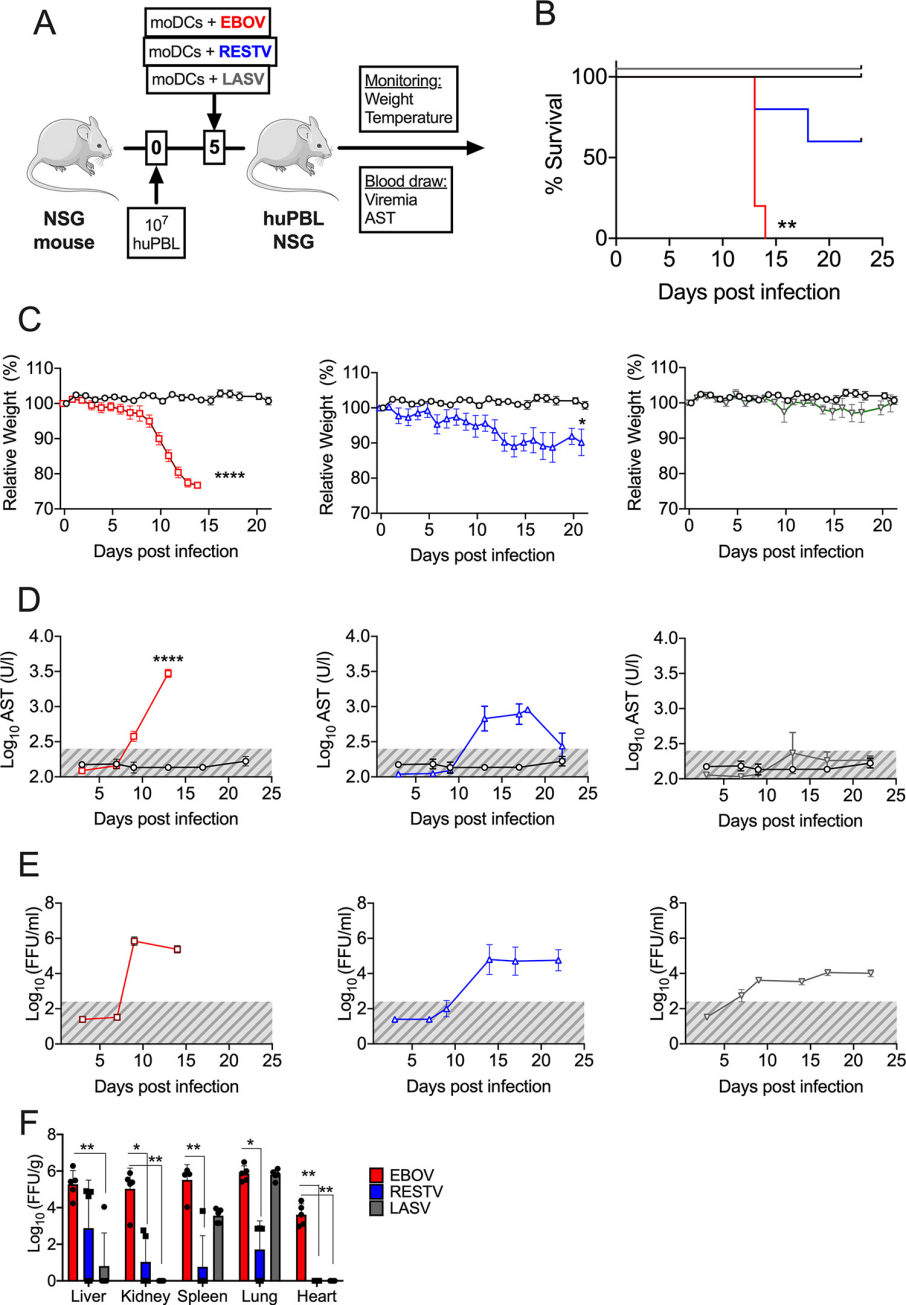
Avatar mice recapitulate EVD but not Lassa fever. (A) Model schematic. EBOV, Ebola virus; RESTV, Reston virus; and LASV, Lassa virus. (B) Kaplan-Meier survival curves of avatar mice transplanted with EBOV-infected moDCs (red), RESTV-infected moDCs (blue), or LASV-infected DCs (green). (C) Body weight loss curves of avatar mice infected with each one of the three viruses. (D) Levels of serum AST at the indicated time points in all three models. (E) Levels of viremia. The gray areas represent the limit of detection of the assay. (F) Virus titers in organs at the time of necropsy. Organ titers were calculated via immunofocus assay as described in Materials and Methods section. Across the figure, significance levels are presented as follows: *, *P* ≤ 0.05; **, *P* ≤ 0.01; ***, *P* ≤ 0.001; and ****, *P* ≤ 0.001. The survival curves were assessed via Mantel-Cox, and the comparison of virus titers in organs was assessed via a Kruskal-Wallis nonparametric analysis. All other analyses were conducted with a two-way ANOVA followed by Dunnett’s multiple comparison test. Data are shown as mean ± SEM.

In a recent study, we demonstrated that NSG-A2 mice engrafted with human hematopoiesis via the transplantation of CD34^+^ human HSCs recapitulated the case-fatality ratios of ebolaviruses in humans after experimental infection (13). In order to test whether this was also the case with avatar mice, we transplanted avatar mice with moDCs infected with either EBOV or Reston virus (RESTV), which is presumably nonpathogenic for humans (18, 19). Moreover, to determine whether our findings were also extensive to other hemorrhagic fever viruses, we utilized moDCs infected with Lassa virus (LASV) to mimic human Lassa fever. As expected, EBOV infection resulted in 100% lethality, but the disease course after RESTV infection was significantly less severe with 3 out of 5 mice surviving infection and showing moderate weight loss. Surprisingly, the LASV-infected avatar mice did not show any signs of disease, including weight loss, and all mice survived the infection (Fig. 4A to C). In agreement with these clinical signs, the EBOV-infected mice showed the highest levels of circulating serum AST. The AST levels peaked at day 12 in the RESTV-infected mice and resolved after day 20 postinfection (Fig. 4D). The levels of viremia were also higher in the filovirus-infected mice (EBOV and RESTV) compared to the LASV-infected mice (Fig. 4E). The virus titers in organs at the time of necropsy indicated higher titers overall in EBOV-infected mice with respect to RESTV and LASV (Fig. 4F). Interestingly, despite the LASV-infected avatar mice displaying viremia as well as high virus titers in the lungs at the time of necropsy, they did not show the presence of elevated AST, which coincided with low levels of virus replication in the liver (Fig. 4D to F). Our findings strongly suggest that avatar mice could be used to test the pathogenesis of other poorly characterized ebolaviruses and that the association between T cell-DC crosstalk and disease is a feature of ebolavirus infection but not necessarily of other hemorrhagic fevers.

### EVD survivor avatar mice are protected against EBOV rechallenge.

We next sought to evaluate whether preexisting EBOV immunity would provide protection against an EBOV challenge in the avatar model. To this end, we first generated avatar mice using huPBLs and matched moDCs obtained from the peripheral blood of EVD survivors.

**FIG 5:**
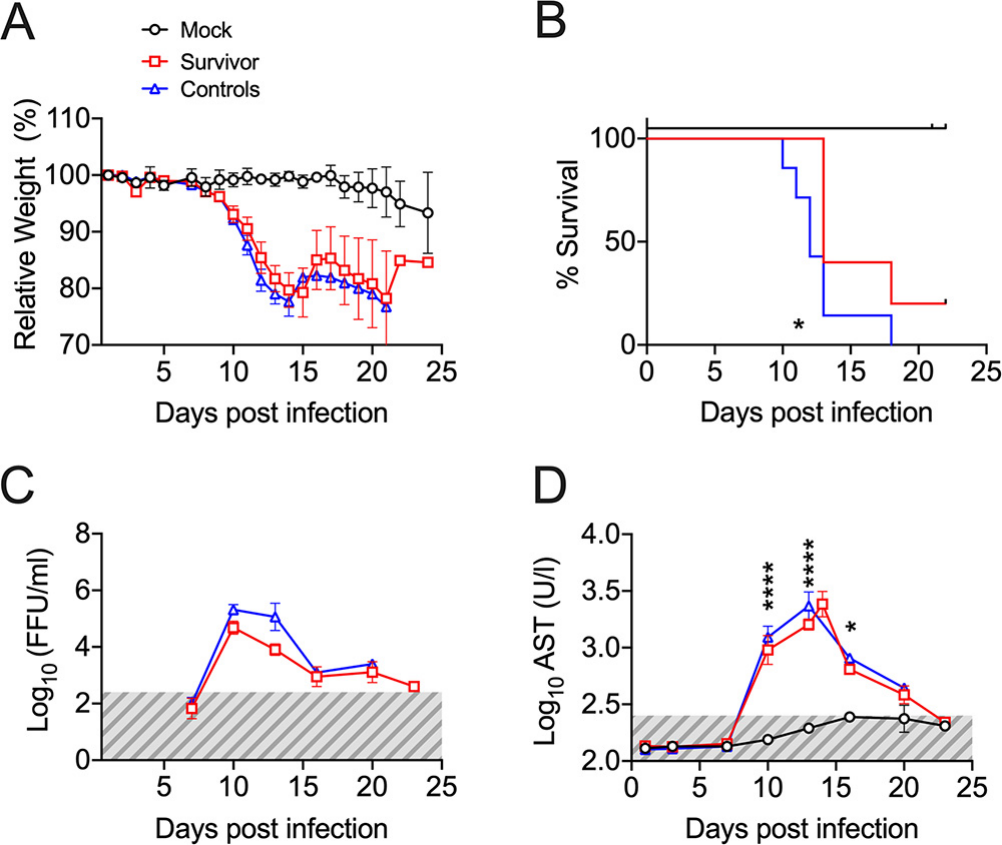
EVD in avatar mice transplanted with survivor huPBLs. (A) Body weight loss curves of avatar mice transplanted with control huPBLs plus noninfected autologous moDCs (Mock, black), control huPBLs plus HLA-matched EBOV-infected moDCs (Controls, blue), or EVD survivor huPBLs plus HLA-matched EBOV-infected moDCs (Survivors, red). (B) Kaplan-Meier curves. (C) Levels of viremia at the indicated time points in survivor-avatars (red) and control-avatars (blue). The gray area indicates the assay limit of detection. (D) Levels of serum AST in survivor-avatars (red) and control-avatars (blue) at the indicated time points. Across the figure, significance levels are presented as follows: *, *P* ≤ 0.05; **, *P* ≤ 0.01; ***, *P* ≤ 0.001; and ****, *P* ≤ 0.0001. The survival curves were assessed via Mantel-Cox, and all other analyses were conducted with a two-way ANOVA followed by Dunnett’s multiple comparison test. Data are shown as mean ± SEM.

Avatar mice engrafted with either survivor immune cells or control cells lost weight rapidly, displayed elevated levels of serum AST and viremia, and succumbed to infection (Fig. 5A to D). Although 1 out of 5 avatar mice engrafted with survivor cells survived infection (Fig. 5B), the overall morbidity and mortality data indicated that the presence of survivor T cells and moDCs did not provide protection to rechallenge. In both the survivor and control avatar mice, we observed the activation of peripheral blood human CD8 T cells characterized by the coexpression of the activation markers CD38 and HLA-DR as well as the reduction of T cell numbers in the peripheral blood, consistent with the activation and migration to infection sites (Fig. S3). Despite T cell activation, our data suggested that T cell mediated immunity was not sufficient to provide protection against EBOV infection.

**FIG 6:**
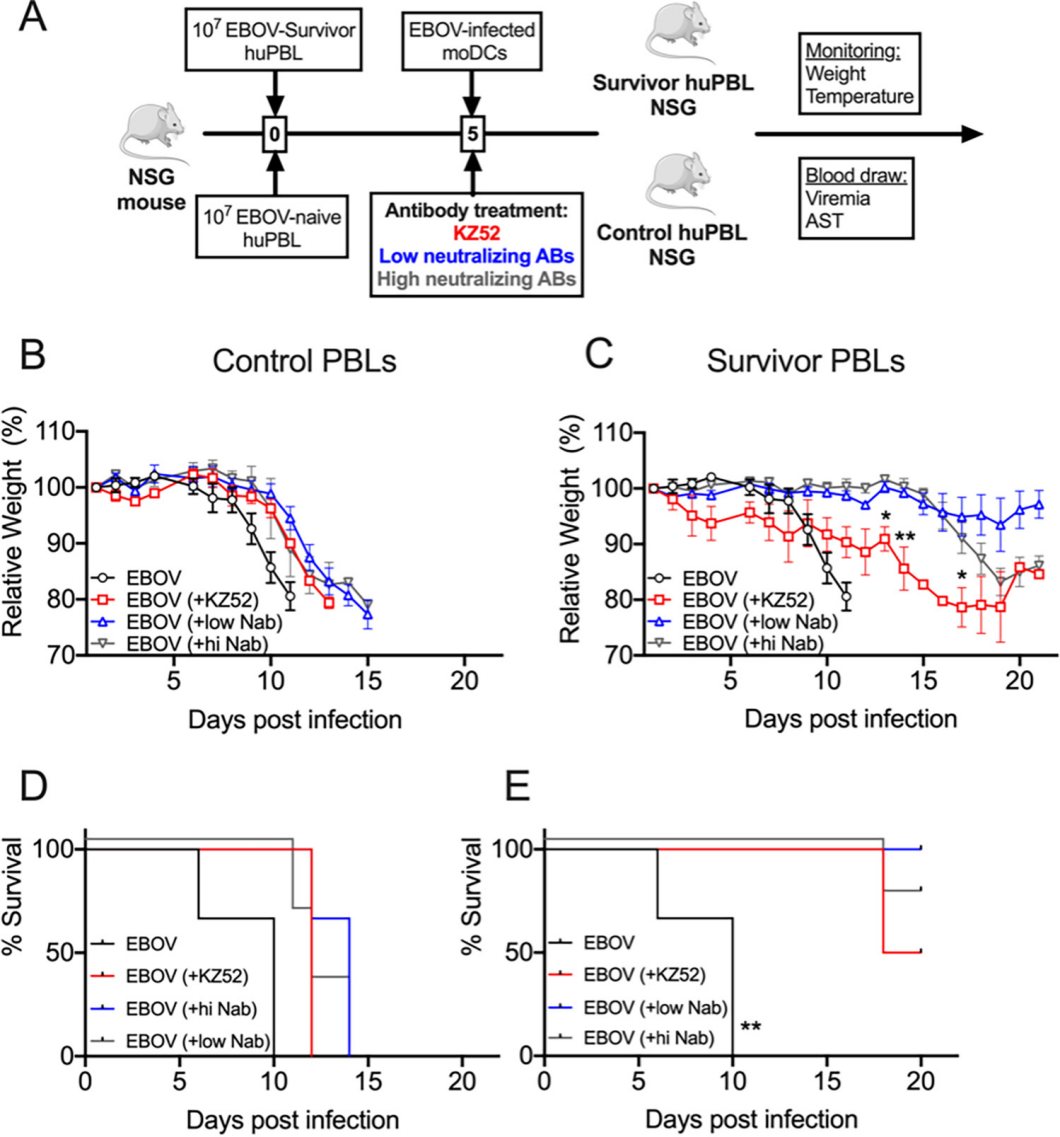
Transplantation of both EVD survivor huPBLs and plasma rescued avatar mice from the EBOV challenge. (A) Model schematic. (B) Avatar mice body weight loss after transplantation with EBOV-infected HLA-matched moDCs in mice that received control huPBLs (EBOV, red), control huPBLs plus anti-EBOV MAb KZ52 (EBOV+KZ52, green), EBOV plus low neutralizing survivor plasma (EBOV+lowNab, blue), or EBOV plus high neutralizing survivor plasma (EBOV+hiNab, pink). (C) Body weight loss curves as indicated in panel B, but in avatar mice that received EVD-survivor huPBLs and HLA-matched DCs. (D) Kaplan-Meier curves of mice that received control huPBLs. (E) Kaplan-Meier curves of mice that received survivor huPBLs. Across the figure, significance levels are presented as follows: *, *P* ≤ 0.05; **, *P* ≤ 0.01; ***, *P* ≤ 0.001; and ****, *P* ≤ 0.0001. The survival curves were assessed via Mantel-Cox, and all other analyses were conducted with a two-way ANOVA followed by Dunnett’s multiple comparison test. Data are shown as mean ± SEM.

We then evaluated whether the transplantation of both T cells and survivor plasma could protect avatar mice from EBOV disease. We set up a protocol in which, together with the transplantation of huPBLs from either the HLA-A2^+^ control individuals or the EVD survivors, mice received plasma from survivors with low neutralizing antibody titers or with high neutralizing antibody titers (Fig. 6A). As a control, a group of mice received 10 mg/kg of the anti-EBOV monoclonal antibody KZ52 (20). In the mice that received control huPBLs and survivor plasma, we did not observe any effect on protection; all of the mice lost weight rapidly and reached the euthanasia criterion in the first 2 weeks after infection (Fig. 6B and D). Notably, the infusion of the KZ52 antibody did not result in any benefit for the EBOV-infected control avatar mice. Conversely, the avatar mice transplanted with survivor huPBLs and matched moDCs survived infection after infusion with survivor plasma or the KZ52 antibody. Of note, 100% of the mice infused with low neutralizing antibody plasma survived infection and showed minimal weight loss, whereas 75% of the mice that received high neutralizing plasma survived (Fig. 6C and E). The transplantation of both survivor huPBLs and plasma also resulted in reduced inflammatory markers, which are characteristic of severe EVD. Luminex data collected at the euthanasia time points indicated that the mice that received control T cells and antibodies showed higher levels of proinflammatory chemokines, such as MIP-1α, MIP-1β, and MCP-1 (Fig. S4), which were previously associated with severe EVD in humans (21).

Taken together, our findings indicate that both T cell mediated responses and antibody responses are required to control EBOV infection. These findings suggest that avatar mice could be used to dissect the relative contributions of both arms of the immune response to infection and to identify correlates of immunity.

## DISCUSSION

Severely immunosuppressed mouse models, particularly the NSG mice transplanted with human immune cells, have been previously used by us and by others to study filovirus pathogenesis in a human-like environment (12, 22). These models recapitulate several important features of EVD, such as viremia, elevated levels of AST, and hypercytokinemia. Indeed, they have been shown to recapitulate the case-fatality rates of ebolaviruses and marburgviruses in humans (13, 23). One caveat of these models is that they typically require the transplantation and engraftment of human hematopoietic stem cells. Thus, the human immune system present in these mice does not retain any memory information. Another caveat is the lack of mature T cells in these models due to the absence of thymic T cell selection (24). However, studying T cell immune responses in the context of EBOV infection is a priority in the field. Clinical immunology data collected in EVD patients during the EVD West Africa epidemic indicate that T cell immunity plays an important role during human EVD (21, 25). In this study, we sought to adapt the NSG model to study the role of T cell immunity in EVD in a human-like environment.

The transplantation of CD14-depleted donor-specific peripheral blood mononuclear cells in NSG-A2 mice resulted in a mouse model in which, at day 28 posttransplantation, up to 60% of the peripheral blood cells were of human origin. Within the T cell compartment, most of the cells acquired an effector memory phenotype, in agreement with previous studies (15). We speculate that this is due, to a great extent, to the absence of thymic output and the lack of stimulation of effector T cell clones, which may lead to reduced naive and T_EMRA_ compartments (26, 27). In agreement with previous findings, the subsequent transplantation of antigen-presenting cells derived from CD14^+^ monocytes resulted in the long-term maintenance of functional T cells in this model (15).

Strikingly, the transplantation of T cells plus EBOV-infected dendritic cells from the same donor in NSG-A2 avatar mice resulted in severe disease and recapitulated important features of human EVD, such as high levels of viremia and virus dissemination, elevated levels of serum aminotransferases, inflammation, and death. These results underscore the importance of the T cell-DC crosstalk in EVD pathophysiology, and they indicate that the presence of these two immune cell subsets alone is sufficient to recall many of the EVD features observed in severely ill human patients. This finding is in agreement with studies in EVD patients and nonhuman primates, which indicated that disease severity was associated with the altered expression of genes related to T cell and DC function (28, 29). This is also consistent with the finding that the dysregulation of T cell immunity is associated with severe EVD (21, 30).

We observed a dose-effect whereby a reduced input of infected DCs resulted in the alleviation of morbidity and mortality in the avatar model. Previous studies have suggested that DCs and resident macrophages are primary targets of EBOV infection at the portals of virus entry (11, 31). Our results are in agreement with the notion that migratory DCs play an important role in EBOV dissemination through their capacity to migrate from infection sites to secondary lymphoid organs. Our data also suggest that both langerin^+^ and DC-SIGN^+^ DCs may contribute to EBOV dissemination.

Despite this role of DCs as “vessels” for infectious EBOV, the transplantation of infected DCs alone was not sufficient to cause disease. Strikingly, a lack of T cells in the system was sufficient to abrogate all symptomatology in the avatar mice, despite high levels of viremia and elevated serum AST. These findings are consistent with clinical data obtained from humans, which demonstrate that severe EVD is associated with high levels of T cell activation (25) and a loss of function in fatal cases (21). Moreover, mismatched TCR-HLA interactions also abrogated disease, indicating that the role of T cells in EVD is dependent on TCR activation by the cognate HLA-peptide complexes presented by the DCs. In this regard, while EBOV survivors tend to mount highly polyclonal T cell responses during the acute phase of EVD, fatalities tend to show a hyperexpansion of a limited number of T cell clones (29). We speculate that, in fatal cases, the inflammation associated with EVD may lead to a reactivation of bystander T cell clones rather than EBOV-specific T cell clones, thereby influencing immunopathology and preventing viral clearance. Bystander T cell activation has been shown in EVD (32, 33) and in other viral hemorrhagic fevers, such as Lassa fever (10).

Regarding the latter, avatar mice engineered with Lassa virus (LASV)-infected DCs survived infection and did not show any symptomatology despite the presence of viremia and virus titers in organs. These results indicate that, despite the described role of T cells on Lassa fever immunopathology (10, 34), other immune cells beyond the T cell-DC crosstalk may be required to recapitulate the severe disease scenario in avatar mice. Indeed, our model seems to be well-suited to study filovirus pathogenesis, but it is likely not translatable to other human infections. The mice generated with RESTV-infected DCs lost weight and showed signs of disease, but 60% of the mice recovered. These findings indicate that the avatar mice reflect, to some extent, the differences in pathogenicity observed between ebolaviruses. This is also consistent with our recent description of the NSG-A2 model harboring human immune cells, in which we demonstrated that the infection of these mice with ebolaviruses recapitulates the case-fatality rates observed for each family member in humans (13).

Innate immune responses likely play an important role in controlling early EBOV replication. However, the main checkpoint for recovery from EVD is likely the efficacy of the adaptive immune response. With this in mind, we sought to determine whether avatar mice could be used to dissect the relative contributions of antibodies versus T cells on EVD survival. The transplantation of survivor T cells alone was not sufficient to rescue mice from death after an inoculation of HLA-matched infected DCs. Perhaps more intriguing was the finding that the passive transfer of a neutralizing antibody, as well as highly neutralizing and low neutralizing survivor plasma, also failed to rescue the mice from death. Conversely, the mice were rescued from EBOV-induced death after the combined administration of survivor T cells plus anti-EBOV antibodies that were either contained in survivor plasma or in the form of anti-EBOV monoclonal antibodies. It is worth noting that the presence of high levels of neutralizing antibodies in the plasma did not provide an advantage over low levels of neutralization in survivor plasma. Previous studies have suggested that effective humoral immune responses against EBOV may require N_AB_ as well as other antibody functions mediated by the Fc receptor (35, 36). Indeed, the current antibody cocktails utilized in postexposure therapy are combinations of neutralizing and nonneutralizing antibodies (37). Previous findings also indicate that a high repertoire of N_AB_, rather than high levels of neutralization, may be required for effective humoral immune responses to EBOV (38). Our findings strongly suggest that both T cells and antibodies are required for protection against an EBOV challenge. This is consistent with observations in EVD survivors that showed high levels of circulating plasmablasts and EBOV-specific T cells (25, 39).

Our findings highlight the importance of humoral and cellular immunity in surviving EVD and also underscore the use of avatar mice to investigate EBOV correlates of protection.

## MATERIALS AND METHODS

### Avatar mice and infection experiments.

The recipient mice were immunodeficient NOD.Cg-*Prkdc^scid^ Il2rg^tm1Wjl^* Tg (HLA-A2.1)1Enge/SzJ (NSG-A2) mice purchased from Jackson Laboratories (Bar Harbor, ME, USA). The animals were housed in individually ventilated cages (IVCs) with autoclaved food, bedding, and acidified water. All of the experiments were conducted with 5-week to 11-week-old males. The mice were inoculated via retro-orbital with 10^7^ CD14-human peripheral blood mononuclear cells (PBMCs) at day 0. The PBMCs were isolated from buffy coats, using density gradient centrifugation to remove residual erythrocytes. Buffy coats from 470 mL of whole blood were diluted with wash buffer (PBS, 2% human serum, 100 U/mL P/S) and layered on 15 mL ficoll gradient solution (Biocoll) in 50 mL polypropylene tubes. Five days after the PBMC transfer, the mice received another retro-orbital infusion of 0.5 to 1 × 10^6^ monocyte-derived DCs from the same donor, which had been either mock-infected or infected with EBOV at an multiplicity of infection (MOI) of 1 for 60 min. Baseline weights were taken on the same day of the DC transplantation. The mice were monitored daily for weight and body scoring. A loss of more than 20% of the initial weight was the main euthanasia criterion. For the evaluation of the viremia and clinical chemistries, the mice were bled at the time points, indicated via the collection of tail vein blood (50 μL). The mice were infected with Ebola virus/H.sapiens-tc/COD/1976/Yambuku-Mayinga (EBOV) (GenBank: AF086833.2), Reston virus (Pennsylvania strain) (RESTV) (GenBank: AF522874.1), and Lassa virus (recombinant Ba366 strain) (LASV). All of the virus stocks were grown on Vero-E6 cells to titers of 10^6^ to 10^7^ focus-forming units (FFU)/mL. Titers were determined via focus formation assays, as described elsewhere. All of the virus stocks were sequenced and certified as mycoplasma free (MycoAlert Mycoplasma Detection Kit, Lonza).

### Generation and infection of monocyte-derived dendritic cells.

To generate the monocyte-derived dendritic cells (moDCs), CD14^+^ cells were isolated from previously harvested PBMCs using CD14 magnetic beads positive selection (Miltenyi Biotec). The enriched CD14^+^ cells were cultured for 5 days at 37°C and 5% CO^2^ in RPMI 1640 (Life technologies) supplemented with 10% human serum, 500 ng/mL gentamicin, 2 mM l-glutamine, 100 ng/mL IL-4, 200 ng/mL GM-CSF, and 2 μM beta-mercapthoethanol. The cells were seeded at a concentration of 3 × 10^6^/mL on 6-well plates. On day 3, half of the medium was replaced with a medium containing cytokines. Cells were harvested on day 5 and evaluated for the expression of markers of immature DCs (CD14^neg^, HLADR^+^, CD11c^+^, CD86^low^) via flow cytometry. The MUTZ-3 cells were obtained from Tanja D. de Grujil (Amsterdam UMC) and were differentiated into DC populations that were enriched in either interstitial DC-like cells expressing DC-SIGN or Langerhans-like cells expressing Langerin, using cytokine-conditioned media as previously described (17).

### Immunofocus assay.

The viremia and organ virus titers were determined via immunofocus assays to determine the focus-forming units (FFU). The blood samples were diluted 1:50 shortly after collection and were stored at –80°C. Single cell suspensions of organs were prepared via mechanical disaggregation using lysing matrix D tubes (MP Biomedicals) filled with Dulbecco’s Modified Eagle’s Medium (DMEM). The homogenization was carried out with a FastPrep homogenizer. For the focus evaluation, 200 μL of serial 10-fold dilutions of organ homogenates and blood were adsorpted into VeroE6 cells seeded on 24-well plates for 1 h and then replaced by a methylcellulose overlay. After 6 days (5 days for the LASV-infected cells), the plates were fixed and inactivated with 4% formaldehyde permeablized with 0.5% Triton X-100 in PBS and blocked with 5% FCS in PBS. The plates were washed with water thoroughly in between steps. The FFUs were detected with a polyclonal mouse anti-EBOV primary antibody (1:5,000) and a secondary peroxidase-conjugated AffiniPure sheep anti-mouse IgG (H+L) antibody (1:10,000). For the final staining, tetramethylbenzidine (TMB) was added, and the foci were counted. For the LASV detection, the cells were incubated with a primary anti-LASV antibody (2F1, monoclonal mouse anti-LASV antibody, 1:20), a HRP coupled secondary anti-mouse antibody (1:1,000), and TMB.

### Clinical chemistry.

The serum samples were diluted 1:10 in water. The quantification of serum aminotransferases (AST) was determined by using commercially available GOT/AST Fuji DRI-CHEM slides in Fujifilm in a DRI-CHEM NX500 analyzer. The limit of detection for AST was 10 U/L.

### Cell recovery and analysis of human cells by flow cytometry.

To prepare the single-cell suspensions of the spleen, lung, and liver, harvested organs were cut into small pieces and digested with collagenase and DNase for 30 min at 37°C. Subsequently, the cells were passed through a 70 μm cell strainer, and the red blood cells were lysed (red blood cell lysis buffer, BioLegend) for 1 min at RT. EDTA blood was lysed for 15 min at RT. After the lysis cells were washed, live dead staining was carried out for 20 min using a Zombie live/dead cell discrimination dye (Biolegend). Then, the cells were blocked for 15 min (Fc Block true stain, Biolegend), stained extracellularly, and subsequently fixed and inactivated (Perm/Fix buffer, BD, with 4% formaldehyde). The samples were acquired with an LSR Fortessa flow cytometer (BD Biosciences) and analyzed with FlowJo software (BD/FlowJo LLC).

### Patients and samples.

The blood donations for mouse reconstitution were provided by the transfusion medicine department at the University Hospital Eppendorf, Hamburg as indicated above. The plasma samples of long-term (6 months to 1 year after recovery) EVD survivors were collected in Guéckédou and Coyah (Guinea) after informed consent was obtained. The plasma and PBMC fractions were processed at Donka Hospital in Conarky and transported to Hamburg in dry ice.

### IgG purification.

Bulk IgG was isolated through affinity-purification using Protein G beads (GE Healthcare) from plasma. The beads were washed three times with binding buffer (20 mM sodium phosphate, pH 7) and incubated with plasma for 2h at RT. The columns were washed with binding buffer, and the incubated beads were applied to the column. After that, the columns were rinsed twice with binding buffer. For the IgG elution, 15 mL polypropylene tubes were filled with 500 μL (10% vol/vol) of neutralization buffer (Tris 1 M, pH 8) and placed under the column on ice. The columns were sealed and incubated for 5 min with 4.5 mL of the elution buffer (0.1 M glycine buffer, pH 2.8). For the final elution of IgG, the seal was removed, and the flowthrough was collected in prepared tubes. The IgG concentration was determined with a Nanodrop system, and the antibodies were stored at –80°C until use. For the antibody transfer studies, isolated IgG with a concentration of 10 mg/kg of body weight was administered i.p. directly after infection, as previously described (40). As a control, anti-Ebola GP clone KZ52 (Absolute Antibodies) were used at the same concentration.

### Statistical analysis.

Statistical analyses were done using GraphPad Prism 6 software. Differences in survival rate were analyzed using the Mantel-Cox test. Differences in weight, AST levels, and serum or virus titers in the blood over time were analyzed using two-way ANOVA, followed by Dunnett’s multiple comparison test. The comparison of virus titers in organs was done via the Kruskal-Wallis test. Significance levels are presented as follows: *, *P* ≤ 0.05; **, *P* ≤ 0.01; ***, *P* ≤ 0.001; and ****, *P* ≤ 0.0001. Data are shown as the mean ± the standard error of the mean (SEM).

### Ethics statements.

All animal experiments in this study were approved by German animal protection authorities (Behörde für Gesundheit und Verbraucherschutz, Hamburg) under the approval N043/2019 and were carried out by qualified staff certified with either category B or C training from the Federation of European Animal Science Associations. Blood donations for mouse reconstitution were provided by the transfusion medicine department at the University Hospital Eppendorf, Hamburg as a leftover product and were approved by the Central Ethics Committee of the German Medical Association. The EDTA-blood samples of the EVD survivors were collected in Guéckédou, Guinea, after written consent was obtained. Samples were collected under ethics protocols approved by both the Guinean National Committee for Research and Health (33/CNERS/15) and by the Medical Ethics Commission of the state of Hamburg (PV5309). The plasma and PBMC fractions were processed at Donka Hospital in Conarky and were transported to Hamburg in dry ice.
