# Supplementary material for: Avatar Mice Underscore the Role of the T Cell-Dendritic Cell Crosstalk in Ebola Virus Disease and Reveal Mechanisms of Protection in Survivors
Source: J Virol. 2022 Sep 8;96(18):e00574-22. doi: 10.1128/jvi.00574-22 (PMC9517696; doi:10.1128/jvi.00574-22)
Supplement: Supplemental file 1 — Fig. S1 to S4. Download jvi.00574-22-s0001.pdf, PDF file, 0.2 MB [file jvi.00574-22-s0001.pdf]

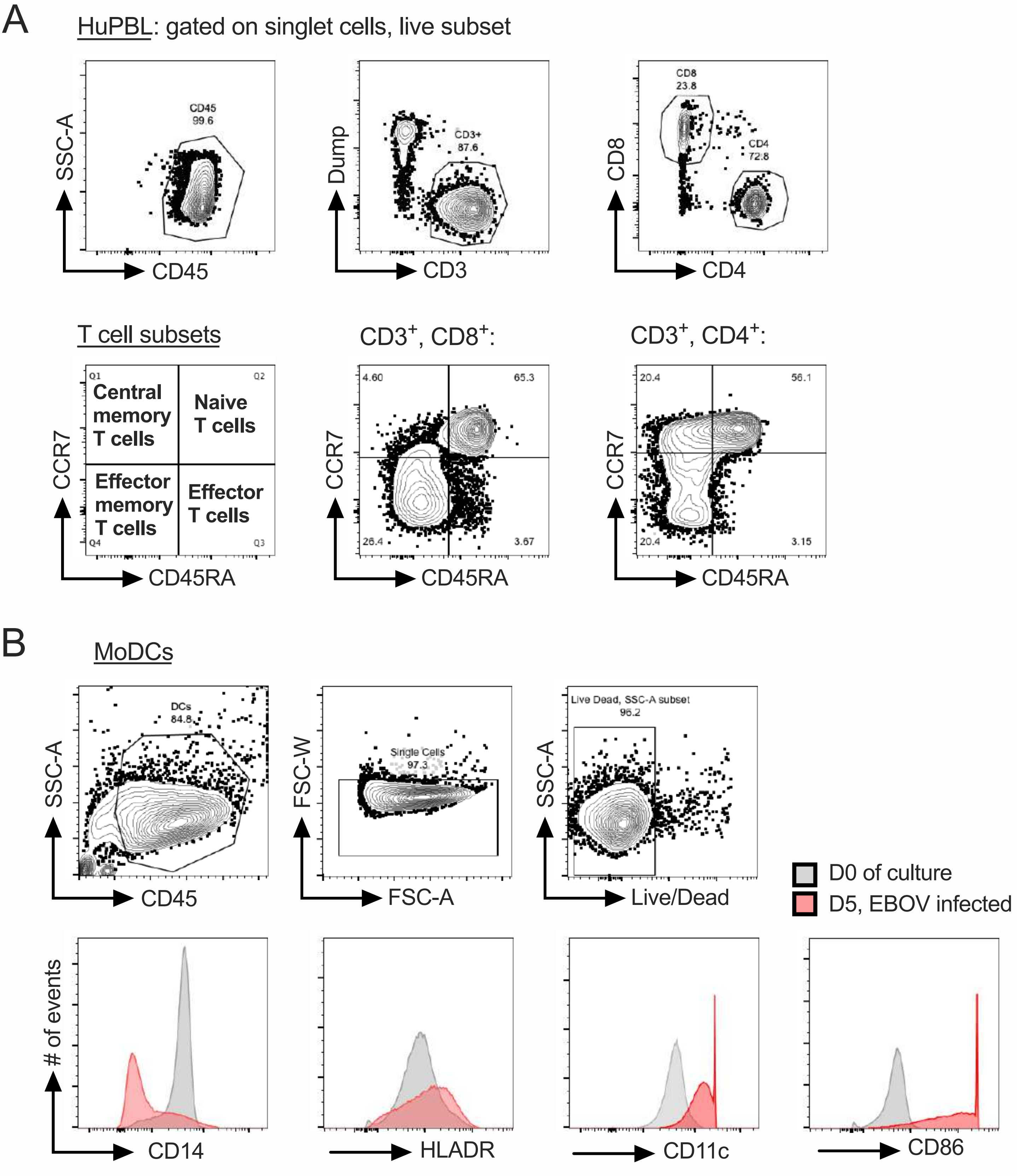

FIG S1 (A) Immunophenotyping of human peripheral lymphocytes (huPBL) after separation from CD14 positive cells. Gating strategy indicates the percentage of CD3 positive cells within the single cell population as well as memory, effector and naïve compartments within CD8 and CD4 T cells. (B) Immunophenotyping of monocyte-derived dendritic cells at day 0 of culture (D0, grey histograms) and five days after infection with EBOV at an MOI of 1 (D5, orange histograms). Histograms indicate transition from CD14 monocytes towards activated CD14 negative/low dendritic cells.

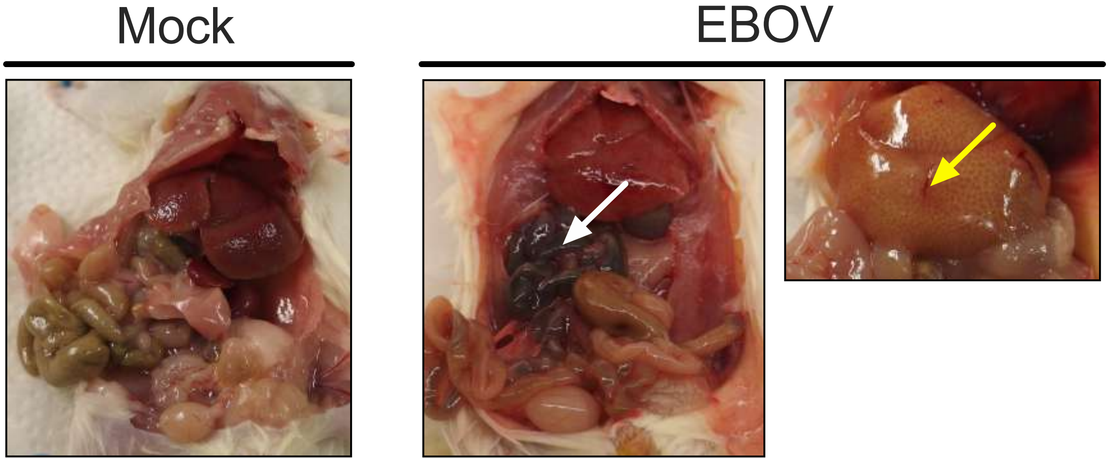

FIG S2 Pathological findings in EBOV-infected avatar mice (right images) and Mock-mice transplanted with non-infected DCs (left). Necropsies were performed in euthanized mice according to the guidelines of our animal experiment protocols. The white arrow indicates gastrointestinal bleeding. The yellow arrow indicates steatosis (fatty liver).

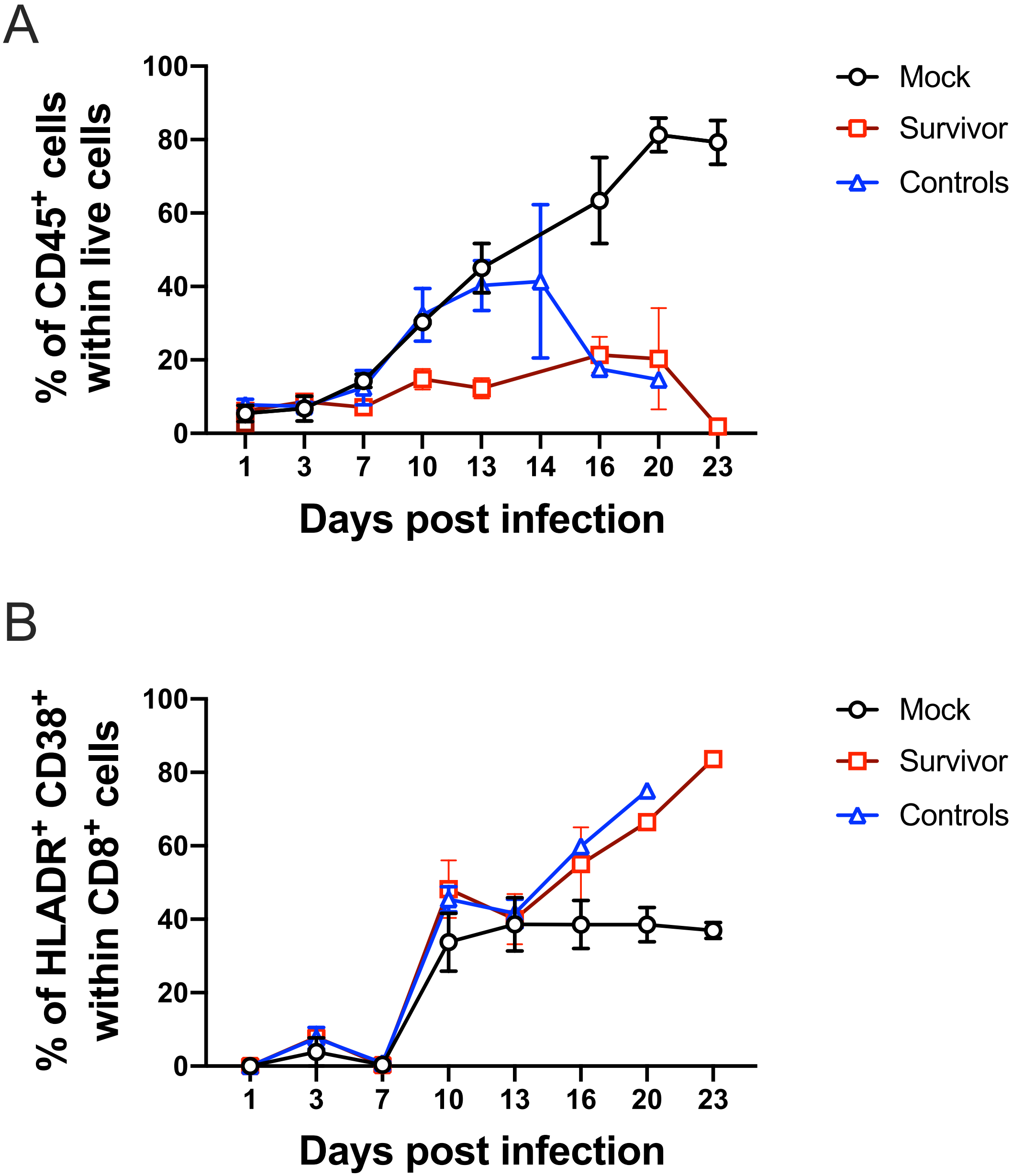

FIG S3 (A) Repopulation of human hematopoietic cells (CD45 positive) in avatar mice transplanted with huPBLs from survivor donors (red) and EBOV-naïve donors (blue). Mock indicates mice transplanted with control huPBLs plus non-infected matched moDCs. (B) Within the CD8 T cell compartment, the graph indicates the percentage of activated CD8 T cells over time, characterized by co-expression of CD38 and HLA-DR. Data are shown as mean ± SEM.

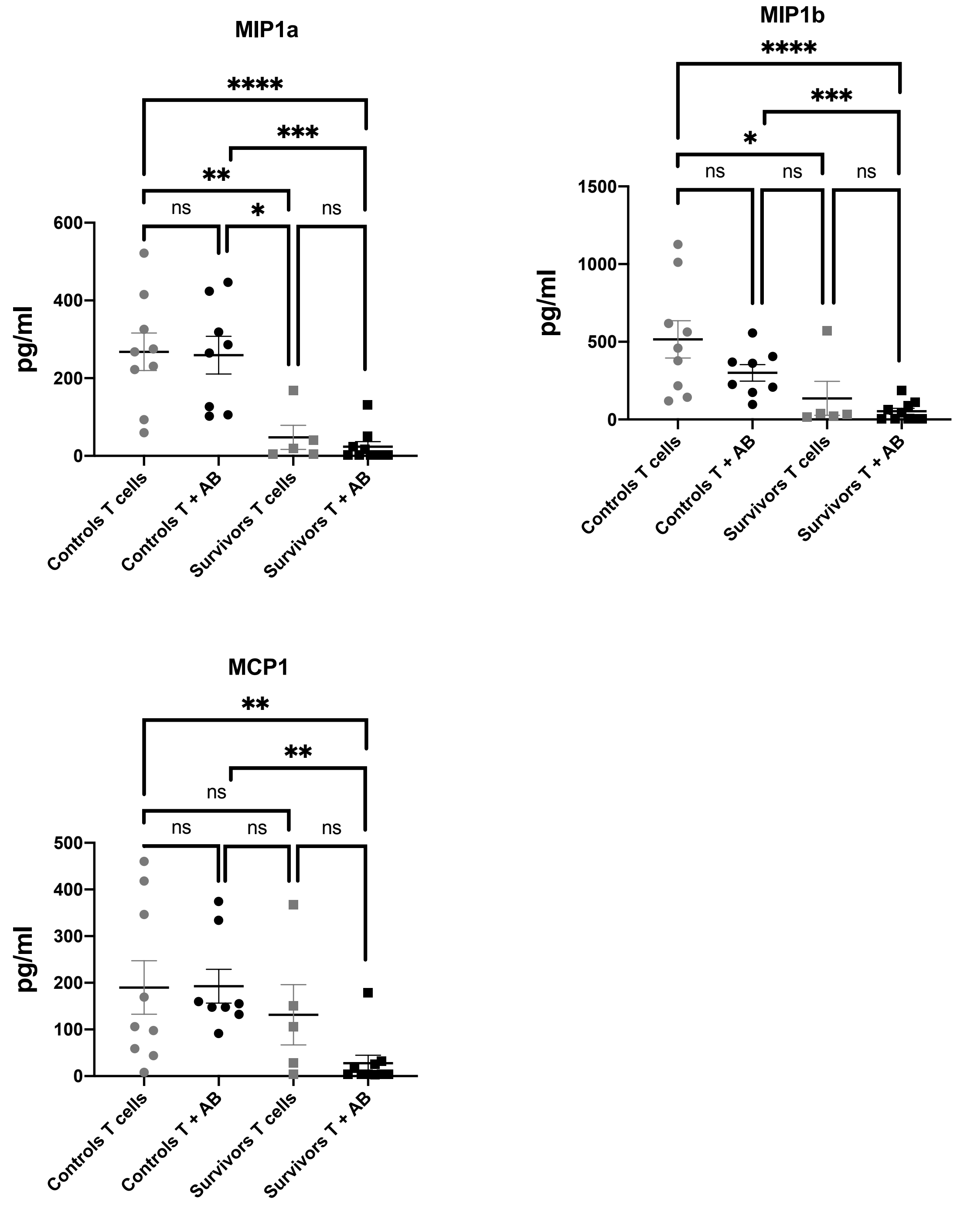

FIG S4 Multiplex ELISA (Luminex) data indicating the levels (pg/ml) of the indicated chemokines in serum at the time of necropsy. Control groups were avatar mice transplanted with EBOV-naïve huPBLs (Controls T cells, n=9) or EBOV-naïve huPBLs plus anti-EBOV serum (Controls T+AB, n=8). Survivor groups were avatar mice transplanted with survivor huPBLs (Survivors T cells, n= 5) or survivor huPBLs plus anti-EBOV serum (Survivors T+AB, n= 9). Across the figure, significance levels are presented as follows: * P ≤ 0.05, ** P ≤ 0.01, *** P ≤ 0.001, **** P ≤ 0.001. Comparisons were done using Mann-Withney non-parametric tests.
